# *WWOX* modulates the gene expression profile in the T98G glioblastoma cell line rendering its phenotype less malignant

**DOI:** 10.3892/or.2014.3335

**Published:** 2014-07-17

**Authors:** KATARZYNA KOŚLA, MAGDALENA NOWAKOWSKA, KAROLINA POSPIECH, ANDRZEJ K. BEDNAREK

**Affiliations:** Department of Molecular Carcinogenesis, Medical University of Lodz, 90-752 Lodz, Poland

**Keywords:** WWOX, glioblastoma, proliferation, microarrays, adhesion

## Abstract

The aim of the present study was to assess the influence of *WWOX* gene upregulation on the transcriptome and phenotype of the T98G glioblastoma cell line. The cells with high *WWOX* expression demonstrated a significantly different transcription profile for approximately 3,000 genes. The main cellular pathways affected were Wnt, TGFβ, Notch and Hedgehog. Moreover, the *WWOX*-transfected cells proliferated at less than half the rate, exhibited greatly lowered adhesion to ECM, increased apoptosis and impaired 3D culture formation. They also demonstrated an increased ability for crossing the basement membrane. Our results indicate that *WWOX,* apart from its tumor-suppressor function, appears to be a key regulator of the main cellular functions of the cell cycle and apoptosis. Furthermore, our results showed that *WWOX* may be involved in controlling metabolism, cytoskeletal structure and differentiation.

## Introduction

Glioblastoma (GBM) is the most common primary brain tumor in adults. It is also the most aggressive and resistant to therapy and has the worst prognosis. To date, there is no effective treatment for patients suffering from this disease (1). After excision of the tumor, its relapse is inevitable, and the mean survival time of patients rarely exceeds several months (2). The molecular pathology of GBM is diverse, and a number of chromosomal aberrations are known to be hallmarks of glioblastoma carcinogenesis. Among them are the loss of heterozygosity on 10q, *EGFR* amplification, *CDKN2A* deletion and mutations of the *PTEN* and *TP53* genes (3). Ineffective treatment, as well as the very high mortality rate and particularly complex molecular background of GBM constitute a strong rationale for research aiming to elucidate the processes underlying the carcinogenesis of neural cells.

The *WWOX* gene is localized at a common fragile site FRA16D (4). It is known to behave as a tumor suppressor. Unlike most tumor-suppressor genes, the loss of functionality of only one of its alleles is enough to predispose a patient to cancerogenesis - the haploinsufficiency phenomenon (5). Growing evidence indicates that *WWOX* is not a classical tumor suppressor. Its action is clearly not limited only to cell cycle control or genome integrity maintenance. Although interactions with several transcription factors and signal transduction proteins are well documented (6–9), it seems that only a tiny piece is known of the physiological cellular role of *WWOX* and its implications for cancerogenesis. Our previous findings on glioblastoma samples showed that the *WWOX* expression level is correlated with genes important to tumor formation and progression, such as *ERBB4*, *Ki67* and *Bcl-2* (10). The present study was conducted on the glioblastoma cell line T98G and aimed to assess the influence of *WWOX* upregulation on the transcriptome and phenotype of these cells.

## Materials and methods

### Cell line and culture conditions

T98G cells, derived from a human glioblastoma, were obtained from the European Collection of Cell Cultures (ECACC). The cells were grown according to the manufacturer’s protocol in Minimum Essential Medium (MEM; Gibco) supplemented with 2 mM L-glutamine (Gibco), 0.1 mM NEAA (Gibco), 10% heat inactivated FBS (Gibco), 0.05 mg/ml penicillin (Gibco), 0.05 mg/ml streptomycin (Gibco) and 0.1 mg/ml neomycin (Gibco) in a humidified atmosphere containing 5% CO_2_ at 37°C.

### Stable retroviral transfection

The *WWOX* gene cDNA was introduced into T98G glioblastoma cells by retroviral transfection. The pLNCX2 retroviral vector with cloned *WWOX* was produced in the PT67 packaging line. Target cells were grown to 30% confluency and infected with the viruses (~10^6^ colony-forming units/ml) suspended in culture medium with Polybrene as vehicle (8 μg/ml, Sigma-Aldrich). After 24 h, the medium was replaced, and stable transfectants were selected with 400 μg/ml G418 (Sigma-Aldrich) for 3 weeks. A pool of stable transfectants was used for the microarray study of global gene expression and biological experiments. Transfection efficiency was confirmed by real-time RT-PCR and western blot analysis.

### Real-time RT-PCR

The real-time RT-PCR procedure was conducted to assess the efficiency of the retroviral transfection and to validate the microarray experiment. Total RNA was isolated using TRIzol reagent (Life Technologies). The cDNA synthesis was performed using 10 μg of total RNA at a volume of 100 μl using ImProm RT-II™ reverse transcriptase (Promega). Reverse transcription was carried out under the following conditions: incubation at 25°C for 5 min and 42°C for 60 min and heating at 70°C for 15 min. cDN A samples were diluted with sterile deionized water to a total volume of 150 μl, and 2 μl was added to the PCR reaction. Real-time RT-PCR was performed using Light Cycler 480 (Roche). PCR products were detected using SYBR^®^ Green I and qPCR Core kit for SYBR^®^ Green I (Eurogentec). All reactions were performed in duplicate. The relative expression levels of the *WWOX*, *BIRC5* and *ID3* genes were assessed. The expression levels of the investigated genes were normalized to 3 reference housekeeping genes (*RPS17*, *H3F3A*, *RPLP0*). The relative gene expression was calculated based on the Pfaffl method (11). Universal Human Reference RNA (Stratagene) was used as a calibrator. The primer sequences, PCR reaction conditions and lengths of the obtained products are available upon request.

### Western blot analysis

Cells were lysed on ice with RIPA protein extraction buffer containing protease inhibitor cocktail (Sigma-Aldrich). Proteins (60 μg) were resolved on 10% SDS-PAGE and were transferred on PVDF membranes. The membranes were blocked for 1 h in 5% non-fat milk and incubated with a primary antibody for 18 h at 4°C. The antibodies used were goat polyclonal anti-WWOX (cat. no. sc-20529), mouse monoclonal anti-ARK1 (cat. no. sc-56881), goat polyclonal anti-KLF8 (cat. no. sc-69294), rabbit polyclonal anti-JAK1 (cat. no. sc-277) (all from Santa Cruz Biotechnology). Subsequently, the membranes were washed with TBST and incubated with the appropriate secondary antibody conjugated with alkaline phosphatase (Sigma-Aldrich) for 1 h at room temperature (RT). Next, the membranes were washed with TBST and developed with Novex^®^ AP Chromogenic Substrate (Invitrogen). GAPDH was used as a reference protein. The relative protein amount was assessed with ImageJ (NIH) based on the integrated density of the bands.

### Microarray transcriptome study

Human OneArray™ (Phalanx Biotech) high-density microarrays were used in flip dye experiments in 4 replicates for each cell variant. Each sample was hybridized against Universal Human Reference RNA (Stratagene) and labelled with ULS™ Labeling Kit (Kreatech Diagnostics). Preparation of the slide for hybridization included prewash in ethanol and pre-hybridization according to the manufacturer’s protocol. Hybridization was performed in a humidity chamber filled with 2 SSPE buffer at 42°C for 16–18 h. Post-hybridization washes were performed with the following buffers: 1 SSPE/0.03% SDS (2 min, 42°C), 1 SSPE (2 min, RT), 0.1 SSPE (rinsed several times, RT). Slide scanning and preliminary normalization were performed with ProScanArray (Perkin-Elmer) and ScanArray Express, respectively.

Further data analysis was performed with the MultiExperiment Viewer (MeV) from the TM4 package provided by The Institute for Genomic Research at http://www.tm4.org/site. For the ontological classification of genes, the PANTHER classification system was used, which allowed a determination to be made of which pathways are susceptible to change depending on the *WWOX* expression level. The microarray results were validated by real-time RT-PCR and western blot analysis. The data have been deposited in NCBI’s Gene Expression Omnibus and are accessible through GEO Series accession no. GSE51481.

### Proliferation, redox potential and apoptosis assays

The three assays assessing cell proliferation, redox potential and apoptosis were multiplexed to eliminate population and culture differences. Proliferation was evaluated with 5-bromo-2′-deoxyuridine (BrdU). BrdU incorporated into DNA during replication was detected with an anti-BrdU monoclonal antibody labeled with europium (Perkin-Elmer). The redox potential was measured with the alamarBlue^®^ cell viability reagent (Invitrogen), the redox indicator metabolized in mitochondria. Apoptosis was assessed by TUNEL reaction with the DELFIA DNA fragmentation assay (Perkin-Elmer). Cells were seeded on a white, clear bottom 96-well plate at a density of 10,000 cells/well. All tests were conducted in a starvation medium (without serum).

### Adhesion assay

In order to assess the ability of the cells to integrate into the extracellular matrix, a colorimetric CytoSelect™ 48-well adhesion assay (Cell Biolabs, Inc.) was carried out. The assay evaluates the capability of cells to adhere to five ECM proteins: fibronectin, collagen I, collagen I V, laminin and fibrinogen. The cells were seeded on a 48-well plate coated with selected ECM proteins at a density of 150,000 cells/well in starvation medium and were allowed to adhere for 90 min at 37°C. Next, the adherent cells were dyed and their number was analyzed colorimetrically.

### Invasion assay

The invasive potential of the investigated cells was evaluated using the colorimetric CytoSelect™ 24-well invasion assay (Cell Biolabs, Inc.). The assay contains a membrane coated with a layer of basement membrane matrix solution and allows for discrimination of invasive cells. The cells were seeded on inserts placed in a 24-well plate at a density of 300,000 cells/well and left to invade for 48 h. Next, the cells that crossed the membrane were dyed and their number was analyzed colorimetrically.

### 3D culture growth

For a three dimensional (3D) culture assay, cells were seeded on a 96-well plate at a density of 15,000 cells/well on a solidified 2-mm layer of growth factor-reduced Geltrex™ basement membrane matrix (Gibco). The cells were grown in an assay buffer consisting of full culture medium and 2% Geltrex™. The assay buffer was exchanged every 4 days. The cells were cultured for 12 days.

### Statistical analysis of biological assays

The results are presented as means. Statistical significance in all biological tests was assessed with the Student’s t-test. The results were recognized as being statistically significant at a confidence level >95% (p<0.05).

## Results

### Retroviral transfection and multiclonal selection allows for stable overexpression of the WWOX gene in T98G glioblastoma cells

**Figure 1:**
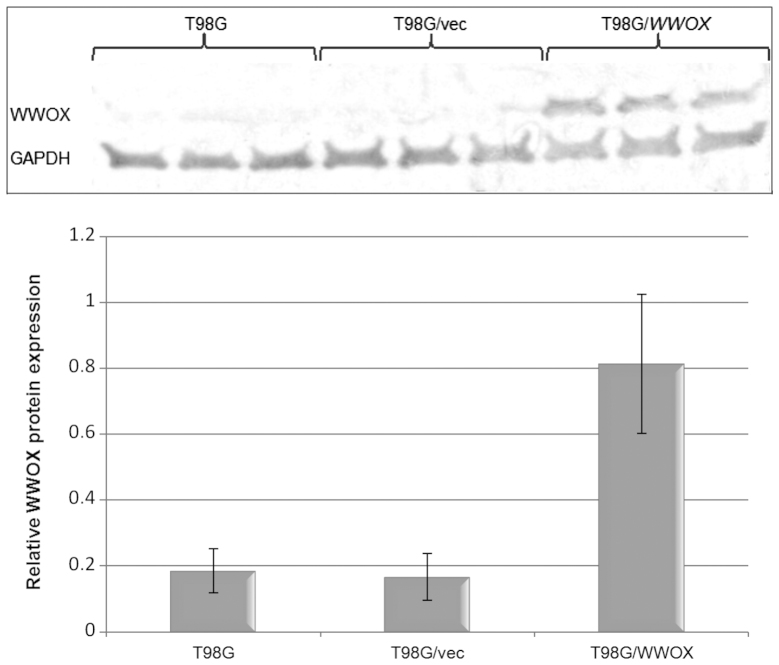
*WWOX* protein levels in the native and transfected T98G cells.

The level of *WWOX* expression was assessed by real-time RT-PCR and western blot analysis. The amount of *WWOX* mRN A in the T98G/*WWOX* transfectants was >29-fold greater than that noted in the T98G/vec control cells. The higher gene expression resulted in an elevated protein level. The protein level in the T98G/vec control was comparable to that found in the untreated T98G cells (Fig. 1).

### Transcriptome analysis of the WWOX transfectants

The microarray study was used to assess changes in the expression level of ~29,000 genes. A total of 2,846 genes showed a significant increase or decrease in expression depending on the *WWOX* level (p<0.05, t-test). For 1,802 of the genes, the change in expression level was 2-fold or greater. All the genes identified in the microarray experiment were ontologically classified using the PANTHER classification system and grouped according to cellular pathways and biological processes. The cellular pathways containing 10 or more genes modulated by *WWOX* overexpression are presented in Table I. Selected biological processes with the highest number of *WWOX*-modulated genes are shown in Table II.

### Phenotype analysis of the WWOX transfectants

To investigate how the changes in the transcriptome translate into cell phenotype, a number of biological experiments were performed. Proliferation rate, redox potential, apoptosis, ability to adhere to extracellular matrix proteins, invasiveness and 3D culture formation were all evaluated in the transfected cells.

### Proliferation, redox potential and apoptosis assays

Multiplexing assays for proliferation, redox potential and apoptosis allowed for elimination of population and culture differences. An analysis of the proliferation rate showed that T98G/*WWOX* cells proliferated 53% more slowly than the control T98G/vec cells (p<0.01). Simultaneously apoptosis was increased, although without statistical significance (p>0.05). A test with alamarBlue to assess mitochondrial metabolism revealed that cells overexpressing *WWOX* had a considerably higher rate of metabolizing the substrate, which may signify an intensification of overall mitochondrial redox potential.

### Invasion

*WWOX*-transfected cells were examined to test whether they exhibit changes in invasiveness. T98G/*WWOX* cells demonstrated a slightly greater ability to cross a membrane coated with basement membrane matrix solution, with 29% more invasive cells. However, the result was not statistically significant (p>0.05).

### Adhesion

**Figure 2:**
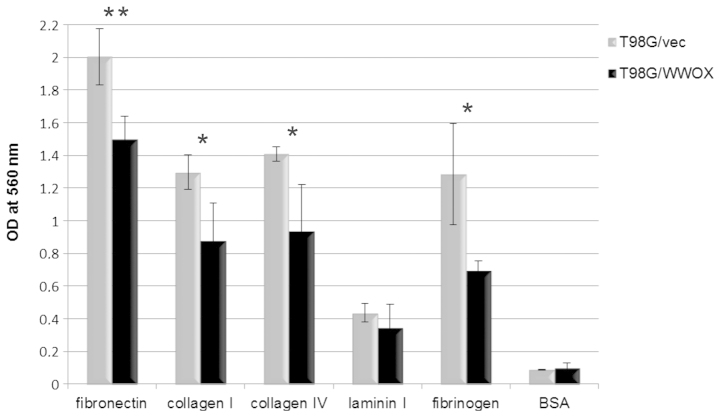
Ability for adhesion of the T98G/*WWOX* and T98G/vec control cells to ECM proteins. ^*^p<0.05, ^**^p<0.01.

Cells overexpressing *WWOX* showed a greatly reduced ability to adhere to extracellular matrix proteins. Decreased adhesion was noted to all examined proteins: fibronectin (p<0.01), collagen I (p<0.05), collagen I V (p<0.05), laminin (p>0.05) and fibrinogen (p<0.05). A graphical presentation of changes in adhesion between T98G cells overexpressing *WWOX* and those with a native *WWOX* level is provided in Fig. 2.

### 3D culture growth

**Figure 3:**
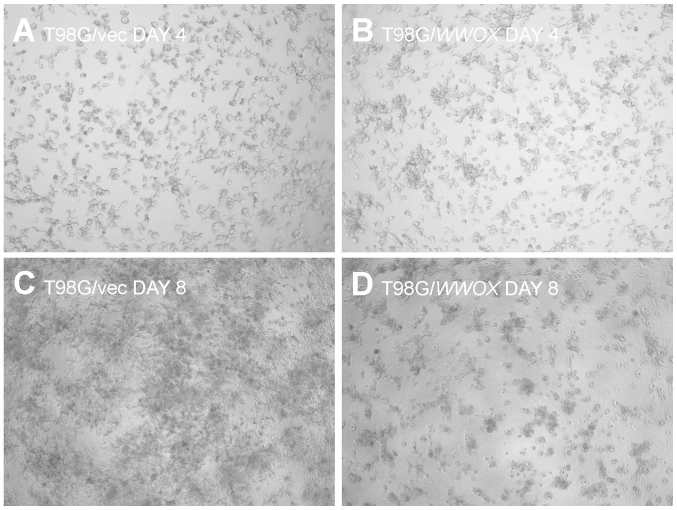
3D culture formation in a Geltrex basement protein matrix. (A and C) T98G/vec cells; (B and D) T98G/WW OX cells. (A and B) 4-day culture; (C and D) 8-day culture.

The T98G/*WWOX* cells exhibited impaired 3D culture formation. The cells seeded in a thick Geltrex^®^ layer after 12 days remained as isolated, single cells that did not proliferate. In contrast, control T98G/vec cells exhibited extensive proliferation and network formation (Fig. 3).

## Discussion

Our previous report showed that *WWOX* may be involved in GBM carcinogenesis and/or tumor progression. The study describes the association of *WWOX* expression with the transcription level of several genes involved in signal transduction and cell cycle control (10). To specify how *WWOX* may influence cancer cell metabolism, we decided to use the T98G glioblastoma cell line, which has a very low level of expression of endogenous *WWOX* to ascertain the effect an increase in *WWOX* expression may have on these cells.

*WWOX* cDNA was introduced into T98G cells by retroviral transfection. In the stable transfectants, the expression profile and basic biological processes were examined. The microarray study for global gene expression allowed the relevance of *WWOX* to be investigated, with regard to overall cellular signaling. The experiment identified 2,846 genes whose expression levels were significantly altered as a consequence of *WWOX* overexpression. The ontological analysis categorized the differentially expressed genes into 121 signaling pathways. The most significant were pathways important both for carcinogenesis and development, such as Wnt, TGFβ, Notch and Hedgehog. The cellular pathways with the highest number of assigned genes regulated by *WWOX* expression level are presented in Table I. The biological experiments on the *WWOX*-transfected T98G/*WWOX* cells revealed a more intensive energetic mitochondrial metabolism, but a lower rate of proliferation and higher apoptosis level than the T98G/vec control cells. Moreover, the T98G/*WWOX* cells demonstrated a higher migration potential and reduced attachment to extra-cellular matrix proteins. Furthermore, cells overexpressing *WWOX* failed to grow in an extracellular matrix environment on Geltrex substrate.

**Figure 4:**
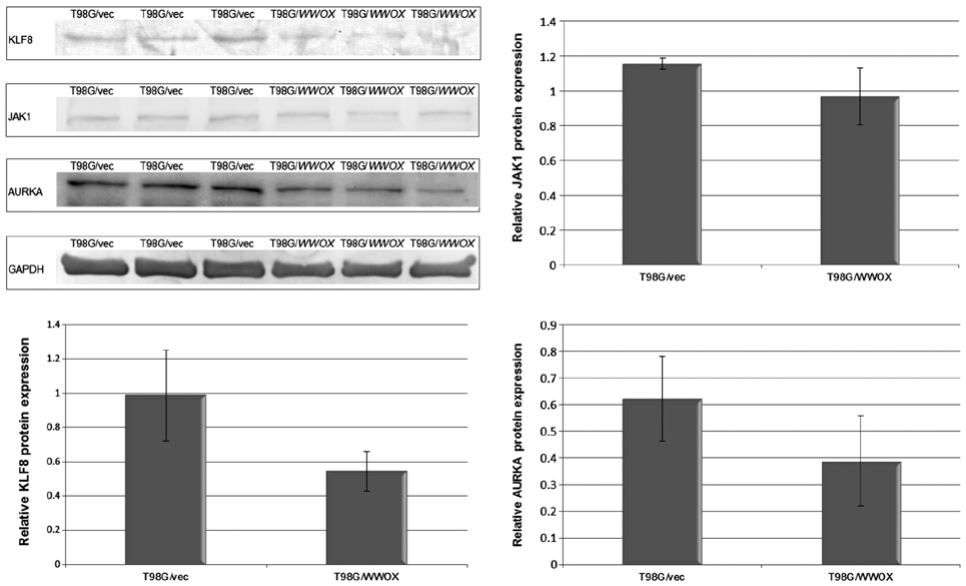
Western blot analysis of the levels of KLF8, JAK1 and AURKA proteins in relation to the *WWOX* expression level. The level of GAPDH was used as a loading control.

Loss of cell cycle control and intense proliferation are hallmarks of neoplastic cells. In the proliferation assay with BrdU, T98G/*WWOX* cells demonstrated a 53% lower proliferation rate than the T98G/vec control (p<0.01). An ontological analysis of the microarray data revealed 163 genes to be involved in cell cycle regulation, whose expression was modulated by *WWOX* overexpression. Among these genes are potent oncogenes linked with brain cancerogenesis: *AURKA*, *KLF8* and *JAK1* (12–16). In our microarray experiment, the T98G/*WWOX* cells demonstrated significantly lower expression of all afore-mentioned genes. Western blot analysis confirmed that the level of these proteins was considerably lower in the cells with high *WWOX* expression (Fig. 4).

After enhancement of *WWOX* expression in the T98G cell line, a tendency for increased apoptosis was observed (p>0.05). Such an effect of *WWOX* overexpression was found to be cosistant across various cell lines (17–24). While apoptosis seems to proceed through a mitochondrial pathway in breast, prostate and lung cells, Chiang *et al* showed that in U373MG glioblastoma cells, *WWOX* overexpression triggers a mitochondrial/caspase-3-independent pathway of apoptosis (25).

*WWOX* overexpression in T98G cells resulted in an increased number of invasive cells crossing the basement membrane. This effect was also observed in breast and colon cancer cells with ectopic *WWOX* expression (26,27). It can be hypothesized that elevated cell motility is related to the function of *WWOX* as a regulator of differentiation. The microarray gene expression analysis revealed a large group of *WWOX-*regulated genes whose products are engaged in nervous system development (108 genes, classification presented in Table II ). Recently, Abdeen *et al* reported that *WWOX* knockdown in MCF-10A normal breast cells resulted in impaired growth in a 3-D culture Matrigel assay and mammary ductal formation (28). This suggests that *WWOX* expression is required for the proper development of this gland. Contrary to Abdeen’s results, in our experiment, the 3-D culture formation by glioblastoma cells was inhibited by a high level of *WWOX*.

The increase in mitochondrial redox activity in T98G/*WWOX* cells is intriguing. One of the genes whose expression was elevated in our microarray experiment was *DLD*, a gene encoding dihydrolipoamide dehydrogenase, diaphorase which catalyzes the reduction of resazurin (alamarBlue^®^) into resofurin (29). The *DLD* expression in T98G/*WWOX* was >3-fold higher than that in the T98/vec control cells. This may explain the higher redox potential observed in the T98G/*WWOX* cells in the alamarBlue assay. O’Keefe *et al* showed in a *Drosophila* model that *WWOX* takes part in aerobic metabolism and the generation of ROS (30). In their recent study, Dayan *et al* claimed that *WWOX* not only influences metabolism, but its mRN A level is also related to the metabolic state of the cell. They reported that switching the metabolism from glycolysis to oxidative phosphorylation causes a stable increase in the amount of *WWOX* mRNA. Consequently, hypoxia, a state where cells rely on glycolysis, causes its decrease (31). GBM cells are known to switch their metabolism to glycolysis in response to hypoxic conditions inside the tumor and the demand for the accelerated production of substrates needed by the rapidly proliferating cells (32).

The T98G cells overexpressing *WWOX* exhibited a significantly lowered ability to adhere to fibronectin, collagen I, collagen IV and fibrinogen. The decrease in adhesion to laminin, was also noted, although without statistical significance. A similar result of *WWOX* influence on decreased adhesion to fibronectin was observed by Gourley *et al* in ovarian cell lines (33). Adhesion to the extracellular matrix is fundamental for cancer cell behavior. Cell adhesion-mediated drug resistance (CAM-DR) is a phenomenon of apoptosis resistance caused by close interactions between cancer cells and ECM proteins (34). Furthermore, one of the main GBM hallmarks is ability for invasion and infiltration of surrounding tissue; this is the reason for the almost inevitable relapse of the disease. The invasion of GBM corresponds with increased adhesion to ECM proteins and its proteolytic degradation (35). In the light of the present knowledge in regards to adhesion in the progression of GBM, the observed reduction in the attachment of cells to ECM proteins caused by *WWOX* overexpression can be interpreted as decreasing cell malignancy.

The decrease in adhesion of the cells to ECM proteins and their reduced ability to proliferate and form 3D structures in the extracellular matrix indicate that *WWOX* overexpression causes substantial changes in cytoskeleton organization and the structure of membrane proteins such as integrins. Indeed, the global gene expression examination showed a large group of *WWOX*-regulated genes acting in the integrin and cadherin signaling pathways (28 and 13 genes, respectively). Moreover, an ontological classification of the genes affected by *WWOX* overexpression with respect to the cellular component revealed that the largest group of genes was connected with the cytoskeleton. The organization of the cytoskeleton regulates such processes as cell adhesion and motility (36,37).

Additionally, besides the cellular functions discussed above, cell cycle control, metabolism, invasiveness, apoptosis and adhesion, several other notable aspects of cell behavior which could be affected by *WWOX* emerged from the microarray study. In the group of genes whose expression was altered by *WWOX* overexpression were genes involved in angiogenesis, development and intracellular transport. The precise mechanism and the nature of *WWOX* acting on the numerous cellular functions indicated here is yet to be determined.

In conclusion, the observed reduction in ECM adhesion, lowering of the proliferation rate and the increase in apoptosis can be regarded as a decrease in cell malignancy due to *WWOX* overexpression. The most striking evidence of the *WWOX* tumor suppressor effect is the inhibition of the growth of glioblastoma cells in an extracellular matrix environment. The higher migration rates may be linked with the presumed role played by *WWOX* in differentiation and the regulation of development.

The obtained results indicate that *WWOX* may be a key regulator of the main cellular processes: cell cycle, apoptosis, metabolism, cytoskeleton structure and differentiation. The increase in *WWOX* expression influences the phenotype of T98G cells, reducing their malignancy.

Present knowledge of *WWOX* function suggests that, contrary to initial assumptions, it does not act as a classical tumor suppressor. It appears that the role of *WWOX* is not limited to that of cell cycle control or genome integrity protection, but its influence on cell function is more global. It is probable that it is one of the pivotal regulators of genes involved in cell differentiation, responsible for maintaining tissue structure.

## Figures and Tables

**Table I:** Ontological analysis of the genes modulated by *WWOX* overexpression: cellular pathways.

Cellular pathway	No. of genes
Unclassified	2,041
Inflammation mediated by chemokine and cytokine signaling pathway	39
Wnt signaling pathway	38
Heterotrimeric G-protein signaling pathway (Gi α and Gs α mediated)	32
Integrin signalling pathway	28
Interleukin signaling pathway	25
Angiogenesis	22
PDGF signaling pathway	21
Huntington disease	21
Alzheimer disease-presenilin pathway	20
p53 pathway	20
Parkinson disease	20
Heterotrimeric G-protein signaling pathway (Gq α and Go α mediated)	20
PI3 kinase pathway	19
TGF-β signaling pathway	18
EGF receptor signaling pathway	17
Endothelin signaling pathway	15
Transcription regulation by bZIP transcription factor	14
Ionotropic glutamate receptor pathway	14
FGF signaling pathway	14
Cytoskeletal regulation by Rho GTPase	14
Apoptosis signaling pathway	13
Alzheimer disease-amyloid secretase pathway	13
Oxidative stress response	13
Nicotinic acetylcholine receptor signaling pathway	13
Metabotropic glutamate receptor group III pathway	13
Cadherin signaling pathway	13
Angiotensin II-stimulated signaling through G-proteins and β-arrestin	12
Ras pathway	11
p38 MAPK pathway	11
T cell activation	10
Insulin/IGF pathway-protein kinase B signaling cascade	10

**Table II:** Ontological analysis of the genes modulated by *WWOX* overexpression: biological processes.

Biological process	No. of genes
Metabolic process	1,063
Primary metabolic process	1,019
Cellular process	777
Unclassified	629
Cell communication	556
Signal transduction	524
Nucleobase, nucleoside, nucleotide and nucleic acid metabolic process	472
Protein metabolic process	415
Transport	370
Transcription	295
Transcription from RNA polymerase II promoter	293
Developmental process	288
Cell surface receptor linked signal transduction	256
Immune system process	243
Regulation of transcription from RNA polymerase II promoter	240
System process	220
Protein transport	192
Intracellular protein transport	192
Neurological system process	176
Protein modification	174
System development	174
Cell cycle	163
Response to stimulus	156
Intracellular signaling cascade	148
Proteolysis	136
Cell adhesion	131
Cell-cell signaling	123
Cellular component organization	122
Ectoderm development	120
Lipid metabolic process	118
Vesicle-mediated transport	117
G-protein coupled receptor protein signaling pathway	114
Nervous system development	109
Mesoderm development	108
Apoptosis	101
